# Targeting Melanin Production: The Safety of Tyrosinase Inhibition

**DOI:** 10.3390/ijms27010097

**Published:** 2025-12-22

**Authors:** Steffen Erler, Ludger Kolbe, Abdulkarim Najjar, Andreas Schepky, Ahmed Kamal, Daniela Lange, Tamara Rogers

**Affiliations:** Research and Development, Beiersdorf AG, 20245 Hamburg, Germany; steffen.erler@beiersdorf.com (S.E.); ludger.kolbe@beiersdorf.com (L.K.); abdulkarim.najjar@beiersdorf.com (A.N.); andreas.schepky@beiersdorf.com (A.S.); ahmed.kamal@beiersdorf.com (A.K.); danieladr.lange@beiersdorf.com (D.L.)

**Keywords:** tyrosinase, Thiamidol, toxicology, safety

## Abstract

Hyperpigmentation remains a persistent dermatological challenge with limited treatment options. Among available strategies, tyrosinase inhibition stands out as the current most effective and safest approach for suppressing melanin synthesis. Thiamidol exemplifies a targeted tyrosinase inhibitor developed over a decade of research, guided by rigorous toxicological evaluation. Although it contains a resorcinol moiety, Thiamidol is not a resorcinol derivative and acts through a distinct thiazole 2-amino moiety. In addition to its targeted mode of action of tyrosinase inhibition rather than inactivation, assessments of cytotoxicity and genotoxicity distinguish it from cosmetic and pharmaceutical active ingredients that form highly reactive ortho- or para-quinones. With further exclusion for off-target effects based on pharma profiling and exposure modeling, Thiamidol’s rapid metabolism and absence of bioaccumulation support its safety profile. While an inhibitory mechanism requires repeated application, this characteristic enhances the safety profile and establishes Thiamidol as a cosmetic ingredient rather than a pharmaceutical. In additional to confirmatory clinical studies for efficacy and skin compatibility, a study shows that the use of Thiamidol-containing products does not negatively impact the dermatological diagnostic accessibility of naevi, which is important for skin health monitoring.

## 1. Introduction

Melanin is a naturally occurring pigment and the main factor responsible for the color of eye, hair and skin. It is synthesized within melanosomes, specialized organelles in melanocytes, by a process called melanogenesis. A variety of melanogenic enzymes are involved during its synthesis, with the last step being the transfer of melanin-containing melanosomes to neighboring keratinocytes [1,2,3,4]. Increased melanocyte activity and melanin production can cause an abnormal accumulation of melanin, leading to hyperpigmentation disorders such as melasma, senile lentigo and post-inflammatory hyperpigmentation [5,6,7]. Although these conditions are not harmful, they can cause psychological and emotional distress for affected individuals [8,9]. As a result, the demand for skin lightening agents that can safely and efficiently address these conditions is increasing.

A variety of treatment options have emerged, encompassing a wide range of approaches that target different aspects of melanin production and deposition. Most of these approaches are based on tyrosinase inhibition. Tyrosinase is a copper-containing glycoprotein and is the rate-limiting enzyme catalyzing the conversion of tyrosine to melanin via the oxidation of dopa to dopaquinone [10]. Downregulation of melanogenesis by this specific enzyme is, therefore, not just very effective but also the safest mode of action as adverse effects can be avoided if an inhibitor specifically targets only this enzyme.

Another approach is the use of scavenging agents that neutralize tyrosinase-produced melanin precursors [11]. However, this approach is quite challenging since tyrosinase has a turnover rate of 140/s, leading to over 12 million melanin precursors that would need to be captured every day. Therefore, while scavenger agents may improve skin tone issues, it is significantly less efficient compared to tyrosinase inhibition in which only one inhibitor molecule is enough to completely block the activity of the enzyme.

Downregulation of tyrosinase expression, e.g., by targeting MITF expression or activation could be another option; however, nuclear factors like MITF are not only involved in melanogenesis but control the expression of proteins that are related to melanocyte proliferation and differentiation [12]. Hence, targeting these pathways should be approached with caution, as it may activate other pathways and lead to undesired side effects.

Melanin synthesis involves oxidation reactions as well as superoxide anions and hydrogen peroxide, which activate melanocytes and upregulate tyrosinase activity. Therefore, antioxidants can be used to treat hyperpigmentation by scavenging free radicals and reducing the oxidative conversion of melanin precursors [13,14,15]. However, antioxidants primarily mitigate excessive stimulation of melanin production, making their effectiveness limited for substantial results. Therefore, they are often used in combination with other ingredients to simply enhance their overall efficacy.

Another approach is the reduction of melanosome transfer from melanocytes to keratinocytes [16]. In hyperpigmentation disorders, this biological process becomes dysregulated or overly stimulated, causing an abnormal accumulation of pigments in the skin’s outer layers. By disrupting this interaction between melanocytes and keratinocytes, it is possible to suppress melanin production and improve the visibility of pigment spots [17].

In addition, epigenetic factors that influence melanin production could be another innovative approach to target hyperpigmentation. Recent research has identified specific epigenetic mechanisms, including DNA methylation, histone modification, and chromatin remodeling, that can regulate the expression of key genes involved in melanogenesis [18]. However, due to the complexity of epigenetic regulation, further research is required to identify potential active ingredients that can effectively modulate epigenetics to control hyperpigmentation.

Although various therapeutic strategies have been explored to manage hyperpigmentation, such as topical agents like hydroquinone, antioxidants, and inhibitors of melanosome transfer, as well as procedural approaches, their limitations remain significant. Hydroquinone, long considered the gold standard [19], is associated with adverse effects including irritation, contact dermatitis, and exogenous ochronosis [20,21,22], leading to regulatory restrictions and bans in many countries [23]. Antioxidants can reduce UV-induced darkening but lack sufficient efficacy to lighten established spots [24], while agents like niacinamide show variable results in inhibiting melanosome transfer [17,25].

Among these strategies, tyrosinase inhibition has emerged as the currently most effective and safest approach for reducing melanin synthesis. It is crucial to distinguish true human tyrosinase inhibitors from compounds that merely act as substrates for human tyrosinase. While true inhibitors directly suppress enzymatic activity and safely reduce pigmentation, substrates may interact with several compounds, thereby triggering adverse effects. Therefore, accurate identification and use of true tyrosinase inhibitors are essential for safe and efficient treatment of hyperpigmentation disorders.

A majority of reported tyrosinase inhibitors have shown limited efficacy, largely because they were tested against mushroom tyrosinase. However, the substrate specificities of mushroom tyrosinase differ significantly from the mammalian enzyme [26,27]. With a high-throughput screening using a recombinant human tyrosinase (hTyr) construct, structural motifs in small-molecule compounds that efficiently inhibit hTyr could be identified; among them, Thiamidol (isobutylamido thiazolyl resorcinol) was discovered as the most efficient inhibitor of hTyr with a half-maximal inhibitory concentration (IC50) of 1.1 μM [28].

In this research article, we explore the role of tyrosinase inhibition as a safe and effective strategy to manage hyperpigmentation, using Thiamidol as an example. Our investigation delves into the efficacy and safety profile of Thiamidol, emphasizing toxicology assessments related to skin tolerability, genotoxicity and cytotoxicity, along with evaluations of off-target effects and exposure assessment.

As a cosmetic ingredient, Thiamidol intends to cause changes in appearance rather than provide a medical treatment. It follows that the toxicological evaluation of Thiamidol has a focused approach on the use of non-animal testing methods.

To supplement the safety evaluation and implications of Thiamidol’s broad spectrum use as a cosmetic ingredient, this research article includes the results of a naevi study conducted with participants using a Thiamidol-containing product. This assessed whether treated naevi experienced changes that could negatively impact dermatological diagnostic accessibility, defined as the ability to identify a naevus as critical for further evaluation in skin health monitoring.

## 2. Result

### 2.1. Toxicology Evaluation

For local toxicity, evaluations for skin and eye irritation show Thiamidol as not categorized as corrosive or irritating to the skin. These are amongst the first and most established non-animal methods adopted by the Organisation for Economic Co-operation and Development [29]. Clinical skin tolerability studies for the final formulation containing Thiamidol provide confirmatory evidence but also information about consumer sensory experiences.

Evaluating skin sensitization remains challenging due to limitations of in vitro systems for determinations of potency [30]. Many ingredients and impurities can be identified as potential skin sensitizers in individual assays [31]. Here, Thiamidol shares a structural alert for sensitization from its meta-hydroxyl groups with resorcinol; however, the lower reactivity of Thiamidol’s hydroxyl groups due to steric hinderance and lowered electrophilicity results in a much-reduced predicted skin sensitization potency. Furthermore, Thiamidol shows complete resistance to oxidation when incubated with tyrosinase [28], unlike resorcinol that may create highly electrophilic intermediates [32]. Through testing and quantitative risk assessment, concentrations in products can be demonstrated as below induction levels, as exemplified with resorcinol [33].

From established in vitro guideline studies, potential genotoxicity and toxic effects at a cellular level can be elucidated. This can be complemented with a weight-of-evidence of any observed indicators of cytotoxicity in multiple assays. For instance, the 3T3 Neutral Red Uptake (NRU) assay, which was initially developed for phototoxicity, can provide an indication for local toxicity in the absence of UV light [34]. Thiamidol shows low toxicity potential in the 3T3 NRU model with a NR50 (IC50 for neural uptake) at 136 μM, with no biologically significant distinction in the presence of UV light (Beiersdorf, internal report).

Beyond evaluations of mode of action, genotoxicity and cytotoxicity, safety assessments depend on the identification and evaluation of the potential for off-target effects. From the pharmaceutical sector, a wealth of tools and experience is available to investigate a wide range of biological activities. Traditionally, these are used for active drug design for early hazard identification to avoid costly non-clinical or clinical studies [35]. Today, many bioactivity assays are commercially offered to cosmetic manufacturers and companies outside of the pharmaceutical field as ‘panels’ for identifying potential toxicities without animal testing, as well as understanding toxicological study results.

**Figure 1:**
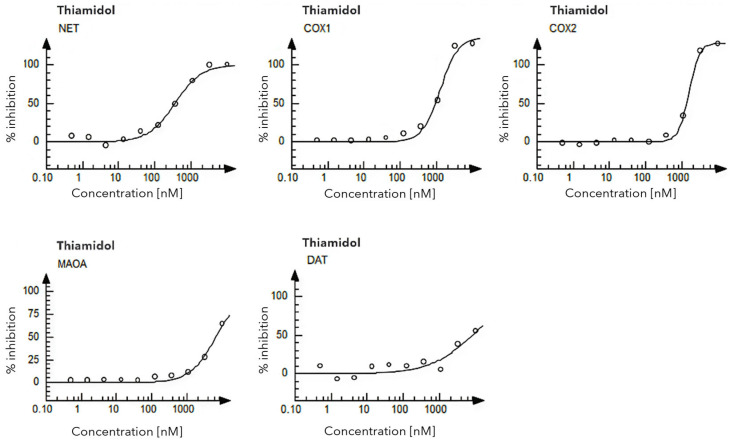
Test dose–response curves for Thiamidol in NET, COX1, COX2, MAOA and DAT assays of the SAFETY*scan*^TM^.

A SAFETY*scan*^TM^ (Eurofins Discovery, San Diego, CA, USA), comprising of 78 assays revealed that Thiamidol has little to no evidence of other specific biological activity. Activity was only evident with an IC50 of 0.36 μM for norepinephrine transport (NET) and >1 μM for enzymatic inhibition of COX1, COX2 and monoamine oxidase A (MOAO), as well as dopamine transport (DAT) (Beiersdorf, internal report) (Figure 1).

The above Figure illustrates how non-animal methods can be used for safety assessment. Further details on tools and methods are available in a large number of publications, with the most prominent case examples being cosmetic ingredients often also cited by regulatory authorities (e.g., [36,37,38,39,40]).

### 2.2. Exposure Assessment

Interpreting toxicology results, especially biological activity from in vitro assays, requires understanding human exposure. Here, consumer use conditions such as quantities, application area or exposure routes, frequency and number of products used are important. However, internal systemic exposure (or dosimetry) also depends on an ingredient’s ability to penetrate the skin and to be distributed to other organs. ‘Physiological-Based Pharmacokinetic’ (PBPK) models developed for pharmaceuticals can be adapted and applied for cosmetic ingredients.

**Figure 2:**
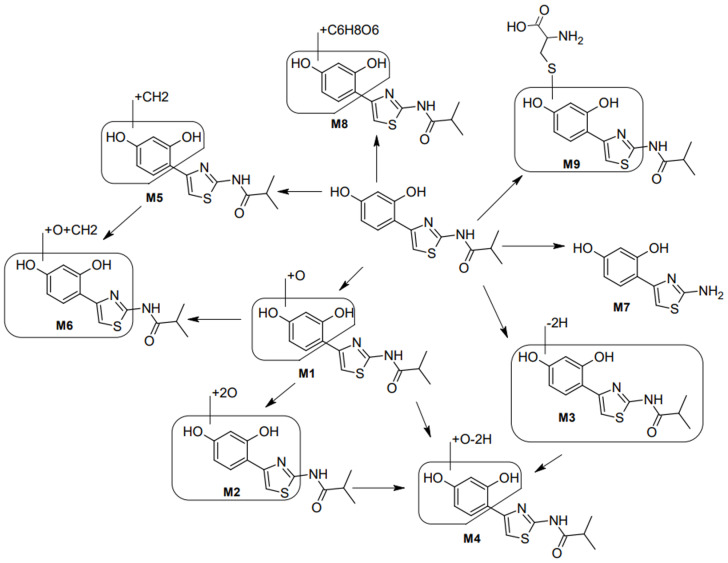
Metabolites (M1–M9) of Thiamidol identified by in vitro skin metabolism. Metabolites M1 (+O) and M2 (+2O) are the result of oxidative reactions at the phenyl ring. M3 formally corresponds to the loss of two hydrogen atoms (−2H), and M4 corresponds to oxidation and twofold dehydrogenation (+O −2H). M5 is the result of methylation at one of hydroxyl moieties. M6 arises from hydroxylation and methylation (+O +CH2). M7 is a hydrolysis product involving loss of isobutyric acid. M8 results from direct glucuronidation, and M9 from S-cysteine conjugation at the core structure. Arrows indicate the metabolic transformations of Thiamidol, showing the sequential formation of downstream metabolites.

A first step for the internal exposure assessment for a topically applied ingredient in vitro requires dermal absorption studies. According to test guidelines, measurements should be made at various levels of skin—the surface, horny layer, epidermis, dermis and receptor fluid [29]. With dermal absorption studies, skin metabolism can be investigated and complemented with studies in other tissues, such as the liver. The analytical identification of metabolites can be facilitated with in silico predictions of metabolic pathways. An overview of the metabolic profile of Thiamidol is presented in Figure 2, with nine metabolites identified (Beiersdorf, internal report).

Metabolism in skin is evident with approx. 50% of Thiamidol degraded in a pig skin assay compared to controls after 24 h, irrespective of test sample concentration. A semiquantitative analysis of the metabolites in the porcine in vitro model is shown in Figure 2, with the primary reactions due to direct glucuronic acid conjugation (phase II metabolism) at one of the free hydroxyl groups. Further degradation products could be attributed to a combination of phase I and phase II metabolism, i.e., hydroxylation and methylation. Consistently, the in vitro study with human hepatocytes demonstrated that Thiamidol undergoes rapid hepatic metabolism, with an in vitro half-life in the minute range, comparable to known high-clearance compounds.

With information on dermal absorption, metabolism, plasma binding and physicochemical parameters, a realistic PBPK scenario can be built. By using PK-Sim and MoBi (Bayer Technology Services, Leverkusen, Germany), a model meeting OECD validation criteria [41], the PBPK profile for Thiamidol was run with several worst-case assumptions, such as no dermal metabolism and no glucuronidation or active secretion in the kidney, which could be refined by considering dermal metabolism and the contribution of kidney elimination (Beiersdorf, internal report). The PBPK modeling intentionally applied worst-case assumptions. This approach was chosen to estimate maximum systemic bioavailability and to ensure a protective safety margin while acknowledging that real-world conditions are unlikely to reflect this scenario. With respect to real-world use, hyperpigmentation disorders such as melasma or hyperpigmentation are not typically associated with a clinically relevant impairment of the stratum corneum barrier that would substantially alter systemic uptake of topically applied compounds with the physicochemical properties of Thiamidol. Thus, the assumption of no metabolism in the skin or kidney can additionally account for variations in skin barrier integrity or systemic clearance, supplementing standard uncertainty factors applied in safety assessment. In this way, even cases of compromised skin can be considered as covered.

To further account for real-world variability and uncertainty, population modeling was performed with body weight and age distributions scaled to reflect the characteristics of the intended target population, ensuring that the model captures a representative range of physiological variability. A PBPK model can be set to cover a 95% confidence level to account for variations in a population and a time period of repeated exposures that results in a blood steady state. For Thiamidol, maximum estimated blood concentrations range from the nM to pM (i.e., parts per billion to trillion), with levels expected below quantification limits of specific analytical methods. The estimated half-life is relatively short, around 1 h, with rapid elimination and limited potential for accumulation in the human body (Beiersdorf, internal report). In addition to conservative assumptions in the model exaggerating exposure levels, results are also worst-case when considering the dermal route of exposure with melanocytes as the target cells, given the body’s mechanisms to shed skin.

Comparing internal exposure metrics from the PBPK model with in vitro SAFETY*scan*^TM^ assay results for Thiamidol shows the potential for systemic exposures at orders of magnitude under IC50 values. This provides a high level of confidence for the exclusion of off-target effects and accumulation in organs. Individual metabolites can also be evaluated to an extent dependent on quantity, structure and physicochemical characteristics. Quantitative-Structure Activity Relationship (QSAR) tools, such as the OECD QSAR toolbox [42], allow for metabolites to be screened for structures of toxicological concern, propensity for organ distribution, and bioaccumulation. This enables a complete picture for the evaluation of an ingredient in terms of bioactivity and metabolic fate.

The importance of exposure assessment and how it guides safety assessment is increasingly supported with recent advancements in toxicological evaluations towards the adoption of internal (systemic) safety values at which no toxicity is expected when genotoxicity and other critical parameters, such as bioaccumulation, are excluded. This follows the body’s innate ability to defend and detoxify, evident from countless toxicology studies across thousands of substances. A concept of interim internal Threshold of Toxicological Concern (iTTC) has been introduced in risk assessment guidance of the European Commission Scientific Committee on Consumer Safety at 1 μM plasma concentration as a conservative benchmark for systemic exposure for cosmetic ingredients [36]. This value was derived for chemicals used in consumer products and is supported by an automated evaluation of ToxCast™ dose–response data, demonstrating that biological activity unlikely occurs below this plasma concentration for non-pharmaceutical active ingredients [43]. The concept has also been developed for specific routes of exposure and classes of substances, such as for inhalation of pharmaceutical intermediates [44] and parenteral exposure to extractables from medical devices [45]. Additional evidence supporting the applicability of this threshold can be found in a recently published case study on several UV filters, which confirmed that systemic exposures for these substances remain well below the proposed iTTC [46]. Consistent with this evidence base, the PBPK simulations indicate that the estimated Cmax of Thiamidol was approximately several orders of magnitude below the interim iTTC.

### 2.3. Implications for Naevi Detection

To support clinical studies and evaluate implications of the use of Thiamidol to naevi detection, a study was performed on the influence of a Thiamidol-containing product (Eucerin Anti-Pigment Spot Corrector) on naevi. Dermatologists evaluated photographic documentation and conducted image analysis to gather data on the naevi’s characteristics. The study comprised a three-month application phase followed by a three-month regression phase. For each subject, the possible effect was compared against a vehicle applied to a second comparable naevus. When comparing both vehicle and verum, regarding the change in skin color a* in either the spot or the surrounding area, no significant differences were found. For both treatments, a significant increase in difference in skin color as well as luminance contrast was observed, with an even more pronounced effect for Thiamidol. Regarding the size of the naevi, no significant differences were found.

**Figure 3:**
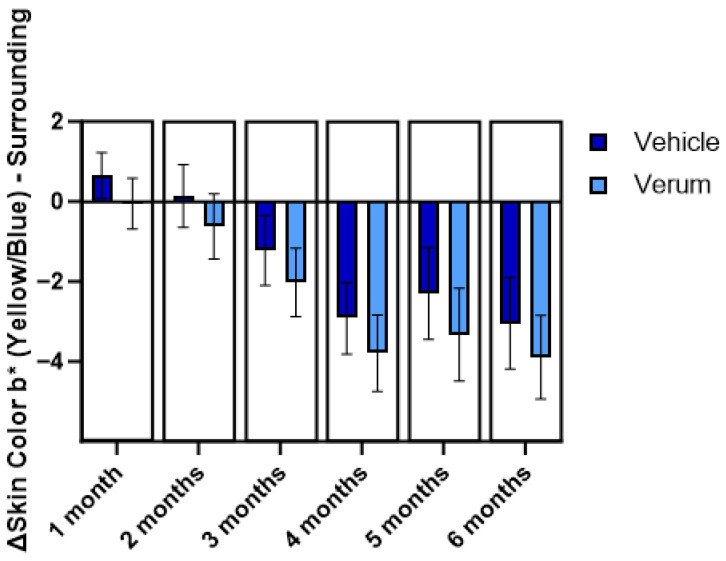
Skin color b* (yellow/blue) by DermLite Image Analysis (DermLite Foto II Pro, DermLite, Aliso Viejo, CA, USA) (of surrounding skin. Bar chart with mean values and 95% confidence intervals of differences to baseline (n = 35–39).

For skin color b*, the study showed no significant differences in the spot area, whereas significantly higher b* values were observed for vehicle treatment in the surrounding area (Figure 3). The observed reduction in the b* value for verum highlights the significant brightening effect of Thiamidol, underscoring its efficacy in diminishing skin yellowness.

Although the test products had a color-altering effect on the naevi, being specifically stronger for the Thiamidol-containing product, the dermatological diagnostic accessibility of the naevi was not negatively impacted, reinforcing the safety of applying Thiamidol while ensuring effective monitoring of skin health.

## 3. Discussion

### 3.1. Toxicological Insights

For an ingredient targeting melanin production, topical application is the best way to reach melanocytes in the outer layers of the skin. The effect on melanocytes does not come via systemic exposure, as with an oral drug. The dermal application with the presence of melanocytes in the epidermis greatly reduces the complexity of exposure assessment compared to an ingredient that needs systemic exposure. The skin is the largest organ of the body, with a high capacity for metabolism and a high barrier function for exogenous substances, so a local application can reduce and even eliminate the potential for significant systemic bioavailability.

With the targeted effect on melanocytes, investigations of melanocyte toxicity and recovery of melanin production in melanocytes provide a sound basis for further investigations. From in vitro and ex vivo studies, the tyrosinase inhibition of Thiamidol differs from inactivation commonly encountered with other substances targeting melanin synthesis, such as resorcinol and catechol [47,48], or melanocyte toxicity in the case of hydroquinone [47]. The IC50 values for cytotoxicity in the 3T3 Neutral Red Uptake (NRU) of 136 μM shows markedly lower toxicity for Thiamidol compared to IC50 values of 17.3, 0.1 and 12.9 μM reported for resorcinol, catechol and hydroquinone, respectively, in the same assay [49].

**Figure 4:**
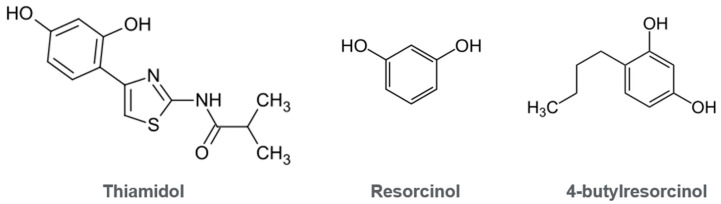
Structure of Thiamidol, resorcinol and 4-butylresorcinol.

When comparing the chemical structure of Thiamidol with resorcinol and its common resorcinol derivatives used against hyperpigmentation, such as 4-butylresorcinol (Figure 4), an immediate distinction should be highlighted. Specifically, Thiamidol is not a resorcinol derivative as it is not manufactured with resorcinol; furthermore Thiamidol cannot be metabolized or degraded to resorcinol. The discovery of Thiamidol identifies its difference as the active group for tyrosinase inhibition: its unique thiazole 2-amino moiety [50].

While Thiamidol contains a resorcinol moiety, its position relative to other functional groups limits the reactivity of the hydroxyl groups compared with resorcinol due to steric hindrance and lowered electrophilicity. The resorcinol moiety in Thiamidol lacks potential for quinone formation unlike catechols and hydroquinone [51,52,53]. In toxicology, depending on the concentration, kinetic rate and site of activity, quinones can be associated with effects ranging from cytoprotection to cytotoxicity and genotoxicity [54]. Hydroquinone, for instance, is associated with a potential to cause DNA damage and interfere with chromosomal separation [55].

In contrast to a pharmaceutical ingredient, a cosmetic ingredient must always be devoid of genotoxicity in vivo, which can be evaluated in well-established batteries of in vitro assays covering both direct and indirect mechanisms, including chromosomal aberration [56]. In the regulatory framework, cosmetic ingredients must be inherently safe. The regulatory framework of an Active Pharmaceutical Ingredient (API) can allow for risk–benefit considerations, as well as warnings such as to not use during pregnancy, common with the medical prescription of hydroquinone.

### 3.2. Exclusion of Mechanisms Linked to Leukoderma and Ochronosis

Exogenous leukoderma and ochronosis have been reported as associated with substrates of tyrosinase or inhibitors of melanin production via other mechanisms. Catechol derivatives have been linked to leukoderma [57], where catechol itself, which was used in cosmetic hair dyes, has been phased out due to local effects of phototoxicity and the potential for oxidative damage [58,59]. Hydroquinone, even though often regarded as the gold standard for the medical treatment of hyperpigmentation, unfortunately has a widely recognized risk for exogenous ochronosis [60]. The most notable cases of exogenous leukoderma are, however, linked to rhododendrol, with its past use in cosmetics in Japan for skin lightening [61,62].

In the case of catechol and hydroquinone, the ortho- and para-dihydroxyl arrangement enable facile two-electron oxidation to quinones, stabilized by resonance. These electrophilic quinones can undergo Michael addition, leading to covalent adduct formation and non-specific enzyme inactivation. Concurrently, the redox cycling between diol and quinone generates reactive oxygen species (ROS) and drives oxidative stress in cells [54]. Rhododendrol adds a clinically relevant twist to this chemistry. Although it is a monophenol, rhododendrol serves as a substrate for human tyrosinase, where it is oxidized into a catechol intermediate, which then undergoes further oxidation to ortho-quinone. This reactive quinone can then act as a suicide inhibitor of tyrosinase, as well as cause oxidative stress and cytotoxicity to melanocytes [63].

By comparison, citations linking resorcinol to exogenous ochronosis originate from a single case published in 1961 [64]. It would be remarkable that exposure to resorcinol can cause ochronosis given its long and widespread use in oxidative hair dyes at up to 5% [65], pharmaceutical applications with concentrations potentially as high as 15% [66,67,68] and its extensive occupational health database [69]. For resorcinol, oxidation with formation of an ortho- or a para-quinone is unlikely, not comparable to catechol or hydroquinone [51,52,70,71]. The meta orientation in resorcinol (1,3-benzenediol) disrupts conjugation necessary to form and stabilize a benzoquinone/semiquinone couple; the meta-hydroxyl groups exert a net electron-withdrawing effect without strong resonance donation to each other, leaving the aromatic ring less activated toward electrophilic oxidation [53]. Consistent with this, voltammetry studies reveal markedly more positive oxidation potentials for resorcinol compared to catechol or hydroquinone [72].

There are no reported cases of exogenous leukoderma or ochronosis from either 4-butylresorcinol or Thiamidol in peer-reviewed journal publications. 4-butylresorcinol is a para-substituted derivative of resorcinol that preserves the 1,3-dihydroxy motif while introducing a linear butyl group at the 4-position. This favorable substitution does not alter the fundamental electronic limitation of the metadiol system, and the butyl substituent further reduces oxidative liability by sterically hindering access to the ring and increasing lipophilicity, suppressing redox cycling under physiologically relevant cellular conditions. Mechanistically, 4-butylresorcinol interacts with tyrosinase as a reversible inhibitor via phenolate coordination to the dicopper center rather than as a substrate, eliminating the electrophilic quinone pathway that drives cytotoxicity in catechols and hydroquinone [55].

For Thiamidol, ortho- or para-quinone formation is chemically and biologically implausible. While 4-butylresorcinol illustrates how simple alkyl substitution preserves the redoxquiet nature of the metadiol scaffold, Thiamidol represents an even more advanced design strategy. Structurally, Thiamidol is much larger and more complex than simple benzenediols: it contains a resorcinol core (a dihydroxyphenyl) that is functionalized with a thiazole ring and an amide group. The presence of the 1,3-thiazole-2-yl amide substituent (attached to the phenyl ring) profoundly alters its chemical and biological behavior compared to catechol and resorcinol and even 4-butylresorcinol.

### 3.3. Further Areas of Research and Limitations

While tyrosinase inhibition affects the amount of melanin synthesized, a potential area for research is into whether tyrosinase inhibition influences qualitative characteristics of melanin. Since tyrosinase inhibition occurs upstream of melanin formation, there is no indication nor research that leads to the assumption that changes in subunit composition, optical properties, photoreactivity and ROS scavenging could occur by blocking tyrosinase activity. Nevertheless, future research could investigate if pigment quality remains unchanged relative to untreated cells and compare impacts with changes in quantity during hyperpigmentation and during treatment.

The naevi study was conducted as a pilot with a relatively small sample size (n = 35–39), which limits the generalizability of the findings. While larger-scale studies with more diverse populations and extended follow-up would further confirm the findings, the current evidence and feedback from dermatologists provides no indication of compromised diagnostic accessibility.

### 3.4. Human Trials and Post-Market Surveillance

Once a safety release is given for human trials, a range of clinical studies can be selected to evaluate skin compatibility ranging from clinical grading to consumer self-reporting, where testing with benchmarks is common practice. The Human Repeat Insult Patch Test (HRIPT) is offered by many dermatological laboratories, although it is not necessarily required for evaluations of skin compatibility (e.g., [36,73]). Importantly, to conform with ethical standards, a HRIPT must have a specific safety release that excludes risks to the participants from the high test concentrations and occlusive test conditions that are usually unrealistic for the intended product use [74].

In recent years, Thiamidol has been tested in several clinical studies to prove its efficacy and skin compatibility across various hyperpigmentation disorders [75]. Research has demonstrated that Thiamidol effectively reduces hyperpigmentation in conditions such as UV-induced solar lentigines [28,76], melasma [77,78,79] as well as acne- and laser-induced post-inflammatory hyperpigmentation (PIH). Notably, clinical studies have reported no significant adverse effects associated with Thiamidol formulations, highlighting its tolerability across diverse skin- and phototypes [80].

In addition, cosmetovigilance and pharmacovigilance require the monitoring and evaluation of undesirable effects from products on the market. In most developed countries, serious adverse reactions, as well as trends in complaint rates, also referred to as signals, must be reported to regulatory authorities. Post-market surveillance also serves to protect consumers and patients from adulterated or even counterfeit products, such as reported for products marketed for hyperpigmentation (e.g., [81,82]).

For products with topical application, cases of contact dermatitis are not uncommon, especially for pharmaceutical products. Dermatitis can often result from irritation, individual predispositions and underlying conditions or co-exposure to other products. Undesirable effects from Thiamidol products are rare and complaint rates are comparable with similar products that do not contain Thiamidol. In particular, there can be intolerances to others ingredient in a formulation, such as alcohols and fragrances. Since the market launch of Thiamidol in 2018, cosmetovigilance data from 50 million products are consistent with observations from more than 120 clinical studies supporting efficacy and skin compatibility (Beiersdorf, internal report).

## 4. Material and Method

### 4.1. Toxicology Evaluation

Thiamidol used for testing was sourced from Alzchem Group AG (Trostberg, Germany) with a purity of 99% *w/w*. For standard toxicological endpoints of skin irritation, phototoxicity and genotoxicity, in vitro studies were performed following equivalent methods to those described in the corresponding OECD Testing Guidelines: OECD Test Guideline No. 431 (In Vitro Skin Corrosion: Human Skin Model Test) [83]; OECD Test Guideline No. 439 (In Vitro Skin Irritation: Reconstructed Human Epidermis Test Method) [84]; OECD Test Guideline No. 432 (In Vitro 3T3 NRU Phototoxicity Test) [85]; OECD Test Guideline No. 471 (Bacterial Reverse Mutation Assay) [86]; OECD Test Guideline No. 487 (in vitro Mammalian Cell Micronucleus Test) [87]; and OECD Test Guideline No. 476 (in vitro Mammalian Cell Gene Mutation Test) [88].

Pharmacological profiling was performed with the SAFETY*scan*^TM^ E/IC50 SELECT service provided by Eurofins Discovery (San Diego, CA, USA), based on the findings of in vitro assays used in pharmaceutical companies [35,89]. Increasing concentrations of Thiamidol (0.1 µM to 10 µM) were tested against various ion channels, and a total of seventy-eight assays performed utilizing various output reads, including GPCR cAMP modulation, calcium mobilization, nuclear hormone receptor interactions, KINOMEscan binding, ion channel interactions, transporter interactions and enzymatic reactions. Methods are available online via Eurofins Discovery in the format of a sample report [90].

### 4.2. Exposure Assessment

A PBPK model for small molecule drugs implemented within the software PK-Sim Version 8.0 (https://www.open-systems-pharmacology.org/, accessed on 18 December 2025) [91,92,93,94] was extended with a model for dermal permeation by Danick et al. [95]. Key inputs are described below.

For dermal penetration, several formulations were tested to assess the dermal absorption and percutaneous penetration of Thiamidol in formulations with differing concentration (0.1%, 0.3% and 1%) through skin layers over 24 h. The methods were similar to OECD Test Guideline No. 428 [96,97]. Excised pig skin disks of split thickness (about 1000 µm) from four donors (two replicates each) were used with a formula application of 4 mg/cm^2^ applied on area of approx. 5 cm^2^. Penetration kinetics were measured with 700 μm skin over 24 h with measurements at 12 defined time points to optimize a permeation model implemented in the PBPK model.

A biotransformation study analyzed the degradation of Thiamidol in fresh pig skin at room temperature after 24 h exposure. A solution of 0.3% and 1.0% was prepared in 2-proanol. From this, about 25 µL of test solution was placed in three vials with 2.5 g of skin, as well as controls without skin or containing skin only. All samples were mixed with 0.5 mL of water, shaken and left open. Determination of the content of the test sample was carried out in parallel. After 24 h incubation, the samples were mixed with 2 mL of acetonitrile, placed in an ultrasonic bath for 10 min (resulting in a stop to any metabolic reactions) and extracted with 50% methanol, then shaken well and placed in an ultrasonic bath and filtered. Metabolites were then identified using liquid chromatography coupled with mass spectroscopy. The in vitro intrinsic clearance and half-life of Thiamidol (at 5 μM) was determined by measuring the substrate depletion of the chemical in incubations with human hepatocytes. Hepatocytes were incubated with Thiamidol at 37 °C, and samples were taken from the incubation after defined time points over 240 min.

The in vitro intrinsic clearances and half-life readouts were expressed as µL/min/10^6^ cells and in minutes, respectively. The clearances were converted into the in vivo intrinsic clearances (CLint, mL·min^−1^·kg bw^−1^) and hepatic clearance (CLhep, mL.min^−1^·kg) based on 120 million hepatocytes/g liver and liver weight of (60 kg body weight) adults [98].

The renal clearance was assumed to equal to the glomerular filtration rate and to be varied among the populations. Based on the relationship between the urinary excretion mechanisms of drugs and their physicochemical properties [99], Thiamidol was considered to be re-absorbed up to 75% of the unbound fraction after the glomerular filtration.

Tissue partition coefficients and plasma protein binding were estimated based on the physicochemical properties of Thiamidol. The physicochemical properties of water solubility and lipophilicity of Thiamidol were experimentally measured. The fractions unbound in plasma (*f*up) of Thiamidol were estimated using a machine learning-based model implemented in ADMET predictor (Simulations Plus, Lancaster, PA, USA). The tissue-partition coefficient and permeability were calculated according to Rodgers and Rowland [100]. The whole-body PBPK model within the software PK-Sim was implemented. The skin model was fitted to the in vitro data and modified to simulate the exposure scenario of Thiamidol to the skin surface, assumed to be washed after 24 h before the next dose is applied, and the skin model was linked to the whole-body PBPK model via an additional compartment delivering Thiamidol to the venous plasma.

Thiamidol was simulated for a repeated-dose exposure over two weeks to ensure that the plasma concentrations reached a steady state. The tested formulations were proposed to be applied to facial skin (area of 565 cm^2^). The daily exposure of Thiamidol was simulated at 9 μg/cm^2^/day in non-volatile emulsions assuming 4.66 g/day/person of a Thiamidol-containing product.

### 4.3. Investigation of Implications to Naevi Detection

This study included 40 subjects (mean age 48.5 ± 9.9 years; 8 male, 32 female) and was conducted over 168 days with a Thiamidol-containing product. Two comparable naevi and surrounding skin on either the upper or lower extremities served as test areas. Test materials were applied twice daily using a spot applicator to each naevus and a 5 mm adjacent skin margin. Standardized imaging was performed using the MacIS-XL system, a tailor-made setup equipped with a Canon EOS 5D Mark II (3Gen, San Juan Capistrano, CA, USA) and a calibrated setup for reproducible macroscopic photography, as well as magnified dermoscopic images captured with a Canon EOS 5D Mark III (3Gen, USA) and DermLite Foto II Pro™ (DermLite, Aliso Viejo, CA, USA) under cross-polarized light. Color calibration was ensured using the X-Rite ColorChecker^®^ (X-Rite, Planegg-Martinsried, Germany). Image analysis assessed CIELAB parameters (L*, a*, b*), color contrast (ΔE), luminance contrast (ΔL*), homogeneity and the size of one selected pigmented spot per test area and surrounding skin.

## 5. Conclusions

Although various strategies have been explored to manage hyperpigmentation, many suffer from limited efficacy, safety concerns or insufficient understanding of the underlying mechanisms. Among these, tyrosinase inhibition has emerged as the current most effective and safest approach for reducing melanin synthesis. This must always be distinguished from tyrosinase inactivation, which can lead to undesirable (toxic) effects.

Engaging a toxicologist in the early development of a novel ingredient is key to avoiding extensive research into an ingredient destined for failure. A toxicologist can advise on the design of an ingredient to avoid certain structures, such as strong electrophiles or nucleophiles associated with skin sensitization, and other chemical structures with a propensity for toxicity or conversion to toxic metabolites. Toxicological evaluations also yield insights into causes for adverse reactions and pathology, leveraging experience developed over decades to ensure the safety of ingredients, where cosmetics must be intrinsically safe but pharmaceuticals can be approved via regulatory processes with knowledge and acceptability of some inherent risks.

Thiamidol took over ten years of R&D development to target tyrosinase without melanocyte toxicity. The availability of human tyrosinase was key to its discovery. In parallel to its development and formulation into cosmetic products, toxicology assays established Thiamidol’s safety profile beyond melanocytes.

With rapid metabolism and without bioaccumulation, Thiamidol presents a case example of an ingredient with specific biological activity without indications of side effects or undesirable metabolites. Furthermore, while dermal exposure is sufficient in the outer layers of the skin, systemic exposure is negligible. The reversibility of Thiamidol’s effects has the drawback of needing reapplications but adds to the safety profile and results in its classification as a cosmetic ingredient.

Thiamidol is not a new ingredient, as it has been on the market for more than seven years. Because Thiamidol contains a resorcinol moiety and even contains the word resorcinol in its cosmetic ingredient name, it can easily be confused as being a resorcinol derivative. But Thiamidol is not a resorcinol derivative, inhibiting tyrosinase via a unique thiazole 2-amino moiety.

Recent reviews consistently show how the clinical safety and efficacy of Thiamidol have been demonstrated in clinical studies covering all skin types. Extensive dermatological and consumer experience establishes Thiamidol as a top-performing candidate against the appearance of hyperpigmentation.

## Data Availability

The data that support the findings of this study are available from the corresponding author upon reasonable request.

## Abbreviations

The following abbreviations are used in this manuscript:

API	Active Pharmaceutical Ingredient
DAT	Dopamine Transport
HRIPT	Human Repeat Insult Patch Test
hTyr	Human Tyrosinase
IC50	Inhibitory Concentration
iTTC	Internal Threshold of Toxicological Concern
MOAO	Monoamine Oxidase A
NET	Norepinephrine Transport
NRU	Neutral Red Uptake
PBPK	Physiological-Based Pharmacokinetic
PIH	Post-Inflammatory Hyperpigmentation
QSAR	Quantitative-Structure Activity Relationship
